# Multivariate analysis in data science for the geospatial distribution of the breast cancer mortality rate in Colombia

**DOI:** 10.3389/fonc.2022.1055655

**Published:** 2023-01-06

**Authors:** Carlos Rubio, Miguel Alfaro, Armando Mejia-Giraldo, Guillermo Fuertes, Rodolfo Mosquera, Manuel Vargas

**Affiliations:** ^1^ Facultad de Ingeniería, Universidad de San Buenaventura, Cali, Colombia; ^2^ Industrial Engineering Department, University of Santiago de Chile, Santiago, Chile; ^3^ Faculty of Engineering, Science and Technology, Universidad Bernardo O’Higgins, Santiago, Chile; ^4^ Escuela de Estudios Industriales y Empresariales, Universidad Industrial de Santander, Bucaramanga, Colombia

**Keywords:** breast cancer, data science, georeferencing, spatial clusters, spatial distribution

## Abstract

This research is framed in the area of biomathematics and contributes to the epidemiological surveillance entities in Colombia to clarify how breast cancer mortality rate (BCM) is spatially distributed in relation to the forest area index (FA) and circulating vehicle index (CV). In this regard, the World Health Organization has highlighted the scarce generation of knowledge that relates mortality from tumor diseases to environmental factors. Quantitative methods based on geospatial data science are used with cross-sectional information from the 2018 census; it’s found that the BCM in Colombia is not spatially randomly distributed, but follows cluster aggregation patterns. Under multivariate modeling methods, the research provides sufficient statistical evidence in terms of not rejecting the hypothesis that if a spatial unit has high FA and low CV, then it has significant advantages in terms of lower BCM.

## 1 Introduction

Modeling and simulation are important decision tools that can be useful for the development of epidemiological monitoring strategies (1). However, since each disease has its own biological characteristics, the models must be adapted to each specific case in order to address real situations (2). According to Diekmann et al. (3), for the study of epidemiological dynamics, scaled mathematical models are initially required in the short and medium term (4–6). Stochastic models are mainly used to take into account the randomness of transmission events (7, 8). Since many epidemics have been quickly controlled, these stochastic processes are accompanied by deterministic models (8). The results of these models provided valuable information to guide public health policies, often on a national scale (9). Authors such as Kang et al. (10) developed a model multivariate analysis to investigate the risk factors of the conversion from mild cognitive impairment to Alzheimer’s disease and predict the time to onset of disease. These authors used the factor analytic technique to comprehensively characterize patients’ cognitive impairment through multiple assessments of cognitive ability. The application of the proposed method shows a high prediction capacity. Also, Cutler et al. (11) used multivariate models as an effective psychometric solution to the variability in classification accuracy of D-KEFS Stroop performance validity cutoffs (performance validity tests during neuropsychological assessments). The results indicate that the multivariate approach to performance validity assessment provides a methodological safeguard against sample- and instrument-specific fluctuations in classification accuracy, striking a reasonable balance between sensitivity and specificity. Saez-Jimenez et al. identified patients at high risk of suffer the atherosclerotic cardiovascular disease. These authors use multivariate predictive models to compare risk functions. The best results were obtained by adding markers such as albuminuria and polyvascular disease. Saez-Jimenez et al. (12) identified patients at high risk of suffer the atherosclerotic cardiovascular disease. These authors use multivariate predictive models to compare risk functions. The best results were obtained by adding markers such as albuminuria and polyvascular disease. Additionally, Lee and Hauskrecht (13) propose a new multivariate predictive model to process time series of clinical events. The model was evaluate using data from electronic health records. The results showed that model leads to improved prediction performance compared to multiple baselines. Also, Withanage et al. (14) developed geographic information system (GIS)-based multivariate analysis model to detect risk hotspots of dengue in the Gampaha District, Sri Lanka to control diseases transmission. The developed model can be used as an early warning tool to explore and identify the current situation of dengue in an area providing valuable insights for healthcare authorities to understand disease propagation patterns and allocate scarce public health resources effectively to prevent impending dengue outbreaks and epidemics.

According to the Constitution of Colombia, the country is made up of 32 regions called departments and a capital district, Bogota, with 51,609,474 population (15). Cancer in the female population of Colombia in 2020 caused 18.32 deaths; among them, 2.91 were due to breast cancer (BC) (16). In the female population, BCM between 2011 and 2019 shows a growing trend, going from 10.2 to 14.1 deaths per hundred thousand women; in 2020 this rate fell to 11.3 deaths per hundred thousand women. Regarding female deaths from cancer, BC caused 15.9% of deaths (16).

Studies on the possible causes that promote the generation of cancerous tumors have advanced worldwide in different areas, however this is not the case for research to detect foci of spatial units with a higher risk of incidence or spread than the surrounding units. Understanding the evolution of the disease in each region and its relationship with neighbouring regions is very useful for defining local containment strategies. The analysis of local conglomerates allows the identification of two types of local nuclei: i) hotspots (high contagion regions) and ii) coldspots (low contagion regions). For this case study, the main contribution of geospatial data science is the visualization of transition zones, which identify regions with a high incidence of BC surrounded by regions of low incidence or vice versa. According to Wang et al. (17), spatial and temporal epidemiology is an emerging research method widely used to study spatial and temporal cancer patterns. Space-time analyzes consider the variation of a phenomenon over time and how it manifests itself in the territory (18). These analyzes provide information to define i) differentiated mitigation strategies at the local or regional level; ii) hospital demand iii) infrastructure needs iv) need for human, technical, and technological resources, etc. According to Kang et al. (19), these models require established criteria of spatial contiguity, that is, a matrix of spatial weights (ω). ω is a mathematical representation of the spatial contiguity structure. It encodes the neighbourhood relationships between the units of analysis and is the basis for a wide variety of statistical analyzes that take geographic structure into account (20). Authors such as Bi et al. (21) propose using a matrix ω of the adjacency queen type, which expresses the neighbour relationship between the spatial units.

Detecting scattering nuclei is very important to understand the current status of cancer and the factors involved in its spread. Different authors have proposed methodologies to assess the presence or absence of a scattering core, spatial autocorrelation (22–24), identification of the scattering core position (25–27), and spatial scan statistics (28–31). Authors such as Cheruiyot (32) used the spatial autocorrelation tool Moran’s global index for the spatial detection of economic clusters. Results from four selected industry sectors show evidence of variable global and localized clustering. Likewise, Chen (33) developed two-dimensional spatial autocorrelation functions based on a similar LISA method as Moran’s index and Geary’s C, using the relative ladder function as a weight function to generate a matrix ω with an offset parameter. Spatial autocorrelation functions can be used to reveal deep geographic information and perform spatial dynamic analysis. Additionally, Kumar and Parida (34) used the Getis-Ord Gi spatial statistical technique and vegetation indices to detect hydroponic crops during the season. The evaluation in hydroponics derived from the Getis-Ord Gi method showed reasonable precision.

Díaz-Casas and García-Angulo (35) reiterate that approximately 80% of women with BC do not present the typical risk factors; therefore, there are actually more factors that have not yet been considered for this pathology. This document considers the importance of filling the knowledge gap in terms of environmental studies of BCM and addressing the issue from the perspective of geospatial data science with an environmental perspective and under the departmental territorial delimitation, including the capital district Bogotá D.C.

The 2020 report of the American Cancer Society analyzed in (36), amply illustrates the epidemiological evidence that relates cancer incidence and mortality to the levels of pollutants suspended in the air (**Supplementary Table 1**). The American Cancer Society report concludes that most of the world’s population currently resides in places where air pollution levels, due to emissions from major sources such as industry, power generation, transportation and domestic burning, considerably exceed air quality guidelines proposed by the World Health Organization (WHO) (36, 37). The report mentions the need for more research related to the effect of air quality on morbidity and mortality for cancers other than lung cancer, especially in developing countries (36). In this order of ideas, this is a pioneering research in the linear geospatial data science considering female BCM under the perspective of environmental variables.

Cancer generally refers to all malignant tumours and has the biological characteristics of abnormal cell differentiation and proliferation, uncontrolled growth, infiltration, and metastasis. Currently, standard clinical treatments for cancer include surgery, chemotherapy, radiotherapy, and immunotherapy (38). Diseases of the mammary gland are divided into benign (non-cancerous) and malignant or BC (39). According to the National Cancer Institute of Colombia (35), benign processes (benign tumor, breast hypersensitivity, inflammation and infection) affect more than 50% of women over 20 years of age and represent 51.6% of the causes of breast surgery. Additionally, 3.2% of benign breast biopsy lesions progress to BC (35).

There are multiple risk factors for the formation of neoplastic pathologies, including mutations of the BRCA1 and BRCA2 genes, high breast density, ductal or lobular hyperplasia, hereditary factors, therapeutic use of RX at an early age, obesity, race, and lifestyle (35). However, what is most worrying is that 80% of women with BC do not present the known risk factors; therefore, there are more risk factors that have not yet been considered (40). According to Van Der Groep et al. (41), between 5% and 10% of BCs are hereditary and up to half of them are the result of mutations in the BRCA1 and BRCA2 genes (42). Mutations in these genes affect the body’s production of the proteins necessary for repairing DNA damage, leaving the body exposed to the generation of tumors. For the early diagnosis of the pathology, educational training on the knowledge of BC and self-examination is important (43).

According to Hanahan and Weinberg (44), the six traits that cancer cells acquire during the development of clinical cancer are: maintenance of proliferative signalling, avoiding growth suppression, resisting cell death, activate invasion and metastasis, allowing replicative immortality, and inducing angiogenesis. Four other traits can be considered: energy dysregulation, evasion of the immune response, promoting inflammation, and genetic instability.

For women over 40 years of age, a clinical breast exam is recommended once a year, along with inspection and palpation as a second step for the early detection of BC. Next, and depending on the medical criteria, is mammography, ultrasound, magnetic resonance imaging, fine-needle aspiration, tru-cut needle biopsy, or mammographic image-assisted biopsy (45).

For Kasper et al. (46), there are clear criteria to classify the evolution stage of the BC and, accordingly, the recommended treatments according to the stage of development i) mammary ductal carcinoma (*in situ*)- wide local excision (excision wide), radiotherapy; ii) operable invasive BC - radical mastectomy, radiotherapy, chemotherapy (depending on the results of lymph node studies); iii) advanced BC - chemotherapy, surgery, radiotherapy; iv) BC in the metastatic stage - there are no known curative treatments, although there are survival and symptomatic palliative treatments.

Crouse et al. (47) used geospatial data to find a correlation between environmental variables and BC incidence or mortality. The findings suggest an association between postmenopausal BC and environmental nitrogen dioxide concentrations. The polluting presence of nitrogen dioxide is directly linked to industrial areas, high levels of vehicle fleet per unit area and use of coal.

The 2018 world cancer statistics, presented by the International Agency for Research on Cancer in its global cancer statistics report, related the distribution of new cases and deaths from BC by region and type of cancer (48). **Supplementary Table 2** shows the mortality distribution for the 10 cancer types with the highest frequency relative to all reported deaths (48).

Among the multiple risk factors not usually considered as determinants in the study of the BC mortality rate, we can mention, among others, the delay in the provision of health services, the timely delivery of drugs to patients, environmental quality, etc. If we observe the temporal evolution of the mortality rate due to BC in Colombia between 2010 and 2020, we find a growth trend until 2020, when it decreases by 2.2/100,000 with respect to the previous year. However, according to **Supplementary Figure 1**, there is an encouraging decrease in the mortality rate from 2014-2020. This slowdown in the evolving mortality could be associated with multiple causes, for example, positive impacts of health sector management, educational training, etc.

Paradoxically, figures from the United States National Cancer Institute, specifically from the annual report to the nation (49, 50), show that, among the 20 types of cancer studied, BC in women is one of the 14 cancers with decreasing mortality trends in the period 2014-2018. Dimensions such as agility in the provision of services and the level of resources allocated to the health sector could favourably impact the mortality rate from BC in developed countries.

A fundamental concept in clinical epidemiology is public health surveillance. The public health surveillance is traditionally defined as the ongoing systematic collection, analysis, and interpretation of health data, which is essential to the planning, implementation, and evaluation of public health practice and is closely integrated with disseminating these data for prevention and control (51). GIS technology software are tools that help monitor health inequalities (52). Other authors, such as Loyola et al. (53), highlight the importance of cross-sectional data with the use of GIS systems to analyze aspects such as spatial auto-correlation, aggregation patterns, cross-correlations, etc., with the generation of dispersion maps and indices that are part of the geospatial data science.

In this document, the first section corresponds to the present introduction, in which, in addition to the motivation of the research, we present the most relevant antecedents in the corresponding line of knowledge, the fundamental elements of cancer biology, in particular BC, BC epidemiology and epidemiological surveillance. In the second section, materials and methods, we refer to GIS and present the elements that make up the research data and their respective sources. We then present the results, discussion and conclusions.

## 2 Materials and Methods

This research is framed within the study of BCM at the level of continental Colombia using official primary sources with data management methodology from the WHO. The estimation of parameters in the modeling stage and their respective diagnostic tests of hypotheses were developed with the implementation of GIS.

### 2.1 Data sources

The set of data on BCM available in Colombia made it possible to undertake the present investigation considering geographical subdivisions of departmental extension. The primary source of information was the web portal of the National Department of Statistics of Colombia (DANE, acronym in Spanish) (15), and the data were processed taking into account the guidelines of the WHO, for the elaboration of the index under study.

The complete statistical data from the research repository are made available to the scientific community (54). The software used was Open Geoga (55). Additionally, the maps were elaborated using ArcGis software (56).

### 2.2 Study population

The physical geography of Colombia is characterized by its enormous diversity. There are flat regions (18%) and mountainous systems (62%) and large depressions (20%). The mountainous systems are found both in the Andean region and in the jungle territories of the southeastern part of the country. The flat regions of the country are composed of valleys and plains. In the large valleys framed by the Andean mountain range, are the places where the main cities are located. In the extensive plains of the eastern part of the country, the population density is very low. In addition, Colombia has 6.4% of the Amazon rainforest, which represents 42% of the country’s area.

The high variability related to geographic location, the range of 0.0 to 21.6 for BCM, the observance of the lowest BCM values in the least populated regions, which are also the most remote from the geographic and productive center of the country, is sufficient motivation to develop the present research, which gives credibility to intuitive statements related to the fact that the effects of geographic location, the regional availability of large forested areas generating good environmental quality and the low intensity of hydrocarbon use, generate competitive advantages in relation to other regions with unfavorable numbers in the same variables.

For the reasons stated and the availability of databases, we decided to consider mortality from BC in light of the territorial distribution of the FA (57) and the CV (58, 59) in each spatial unit regarding the national total for a cross-section from the 2018 census.

The island of San Andres is not part of the study because in the geospatial contiguity analysis a Queen matrix was used, and in this type of contiguity matrix the contiguity criterion is based on sharing some point, either edges or corners. The mortality rate due to BC on the island of San Andres will be analyzed in a later study.

### 2.3 Exploratory analysis and moleling

GIS technology is designed to work with information organized in georeferenced databases. These bases make up a spatial analysis unit through geographic coordinates, allowing representation on maps. These maps are analyzed under specific operations and functions (60). GIS integrates common database operations such as querying and statistical analysis with the visualization and geographic analysis offered by maps (61). These versatilities distinguish them from other information systems since the events can be explained and the strategies can be planned and adapted to the geographical characteristics of the units under study.

With a GIS, spatial autocorrelations and cross-correlations between variables can be sought, inferences can be made, and hypotheses contrasted. Together, this provides a scientific character to studies of the spatial distribution of a pathology. In Colombia, the geographic information system for planning and territorial ordering (SIGOT, acronym in Spanish) is the GIS for national territorial planning, and is under the administration of the Agustín Codazzi geographic institute (IGAC, acronym in Spanish) (57).

Moreno-Serrano and Vayá-Valcarce (62) emphasize that the very nature of cross-sectional data is spatial (data from different spatial units located at a given moment). These authors affirm that when working with this type of data, the spatial effects of heterogeneity and spatial dependence often appear. For geospatial research, there are specific information management techniques.

In this study, the rate BCM was calculated following the international guidelines proposed by WHO. Specifically, we considered the BCM as the number of female deaths from BC per 100,000 women in the population. Based on information from DANE- vital statistics (63), we determined Colombia’s annual BCM (**Supplementary Table 3**).

Following the methodological guidelines of Rubio-Sánchez’s research (54), the exploratory data analysis with univariate and bivariate methods, with scatterplot and boxplot generation, allows for visualizing outliers, hotspots, coolspots and the types of correlation existing between the variables. As a result of the exploratory analysis, we proposed a working hypothesis inspired by the behaviour of the variables under study. We explored different models or specifications in the modelling stage to compare the behaviour hypothesis between the variables, among which the one with the greatest likelihood was determined.

### 2.4 Diagnostic testing

Multiple theoretical developments revolve around quantifying a phenomenon at the geospatial level. Regarding indices that quantify dispersion (64), stands out since it extensively exposes the univariate and bivariate Moran index and the Getis-Ord G indicator, among others. The mathematical expression of the univariate Moran index for a variable *X* is described in equation 1.


(1) $$I=\frac{N}{S_{0}}\frac{\sum_{i=1}^{N}\;\sum_{j=1}^{N}\omega_{ij}(X_{i}-\bar{X})\;(X_{j}-\bar{X})\;}{\sum_{i=1}^{N}\;{\left(X_{i}-\bar{X}\right)}^{2}}$$

Where *N* is the number of analysis units, *ω*=(*ω_ij_*) is the contiguity matrix in which *ω_ij_* represents the contiguity weight. $S_{0}=\sum_{i=1}^{N}\sum_{j=1}^{N}\omega_{ij}$ weights for the sum of the entries that make up the contiguity matrix. In the Queen-type weight matrix, there is contiguity if edges or corners are shared; that is, there is at least one point in common, as described by equation 2.


(2) $$\omega_{ij}=\{\begin{array}{c} 1,\;i\neq j\;\\ 0,\;i=j \end{array}$$

However, the weight matrix that describes the contiguities of each spatial unit can be built based on different criteria than the one described in the Queen.

The bivariate Moran index is calculated using equation 3.


(3) $$I=\frac{N}{S_{0}}\;\frac{\sum_{i=1}^{N}\sum_{j=1}^{N}\omega_{ij}\left(X_{i}-\bar{X}\right)\left(Y_{i}-\bar{Y}\right)}{\sqrt{\sum_{i=1}^{N}{\left(X_{i}-\bar{X}\right)}^{2}}\sqrt{\sum_{i=1}^{N}{\left(Y_{i}-\bar{Y}\right)}^{2}}}$$

To determine the existence of spatial autocorrelation, establishing the existence of clusters of study units with high values (hotspots) or low values (coldspots), the Getis-Ord global indicator G is used, which is calculated using equation 4.


(4) $$G=\frac{\sum_{i=1\;}^{N}\sum_{j=1\;}^{N}\omega_{ij}X_{i}X_{j}}{\sum_{i=1\;}^{N}\;\sum_{j=1\;}^{N}X_{i}X_{j}}\;;\;i\neq j$$

In this index, the condition *i ≠ j* is imposed, which means that no unit can be related to itself.

The goodness of fit or coefficient of determination *R* ^2^ is the ratio between the variance of *ŷ* which is estimated using the variance of . The variance is the sum of the square of the residuals divided by the number of observations; that is, $\sigma^{2}=\frac{\sum_{i=1}^{N}{\left(y_{i}-{\bar{y}}_{i}\right)}^{2})}{N}$ , where *N* is the size of the population. When the variance is estimated using a sample of size *n*, the unbiased estimator is: ${\hat{\sigma }}^{2}=\frac{\sum_{i=1}^{n}{\left({\hat{y}}_{i}-{\bar{y}}_{i}\right)}^{2})}{n-1}$ .

In the ordinary least squares (OLS) model, it must be true that: *E(*u*|*Vn_i_* )=0, the mean of the errors regarding the explanatory variables is zero. *Var(*u*|*Vn_i_*)=*σ* ^2^, the variance of the errors regarding the explanatory variables coincides with the population variance (homoscedasticity-equal dispersions). The white heteroskedasticity test contrasts the null hypothesis *H*_0_: In the OLS model, the principle of homoskedasticity is fulfilled, that is, *Var*(*u*|*Vn_i_*)=*σ* ^2^ regarding the alternative hypothesis of heteroscedasticity.

## 3 Results

The BCM in Colombia for the year 2018 was 13.9, meaning 14 deaths on average per hundred thousand women. At the level of the spatial units studied (32 regiones contimentales), the regions with the lowest mortality are on the outskirts of the country: Chocó, Guajira, Casanare, Putumayo, Amazonas, Guainía, Guaviare, Vaupes, Vichada. In contrast, the departments with the highest mortality are Valle del Cauca, Risaralda, Atlántico, Caldas, Quindío and Santander. The map shows that the BCM is not randomly distributed and that, on the contrary, there is an aggregation pattern between spatial entities with some hotspot clusters and other coldspots towards the peripheries (**Supplementary Figure 2**).

The observations support the proposal of a working hypothesis to implement the Getis-Ord general hotspots/coldspots clustering test to detect clustering trends with scientific evidence. Said hypothesis in statistical terms is the null hypothesis, Ho: “There is neither a low-low nor a high-high clustering of the grouping of territorial entities according to the distribution of the BCM variable, spatially lagged regarding itself”.

While for Moran’s global index I, the null hypothesis is Ho: “The distribution of the BCM variable, spatially lagged regarding itself, is random”.

Implementing a matrix of Queen-type neighbourhoods in which the territorial entities that share borders or corners are considered neighbours. The following results were obtained from the Getis-Ord and Moran tests when using ArcGis^®^ (**Table 1**).

**Table 1:** Getis-Ord general G test of hotspots/coldspots clusters and Moran’s global index I of random spatial distribution for BCM in Colombia-2018.

	Observed	Expected	Variability	Z Score	P value
Getis-Ord general G	0.000002	0.000001	0	4.787320	0
Moran’s global index I	0.473835	-0.032258	0.15773	4.029765	0.000056

In the Getis-Ord test, the null hypothesis was rejected, and the existence of high-high clusters in the BCM variable is confirmed. In the Moran test, the null hypothesis was also rejected and concluded that the data distribution pattern is aggregated. Similarly, the spatial exploratory clustering analysis was carried out for the FA index variables and CV. The spatial autocorrelation estimates and inferences for the FA and CV variables are presented in **Table 2**.

**Table 2:** Inferential statistics for autocorrelation and FA and CV clusters.

	Variables	Observed	Expected	Variability	Z Score	P-value	Inference
Getis-Ord general G	FA	0.038231	0.032258	0.000003	3.215022	0.001304	There is sufficient statistical evidence to affirm that the values of FA and CV are distributed in the spatial units in an aggregate manner, forming clusters mainly of units of high values with units of high values.
Moran's global index I		0.586885	-0.032258	0.012827	5.466814	0	
Getis-Ord general G	CV	0.047989	0.032258	0.000039	2.503266	0.012305	
Moran's global index I		0.213581	-0.032258	0.010922	2.352392	0.018653	

Variables FA and CV- Queen type neighbourhood matrix: criteria edges and corners.

The decision rule is to reject Ho, if the pseudo p-value found is less than 5%. In such a case, if the z-score is greater than zero, then the observed G is greater than the expected G, indicating high clustering. Meanwhile, if the z-score is less than zero, then the observed G is less than the expected G, which is an indicator of low-low clustering. The BCM database (cross-section for 2018) is presented in **Supplementary Table 4**.

**Figure 1:**
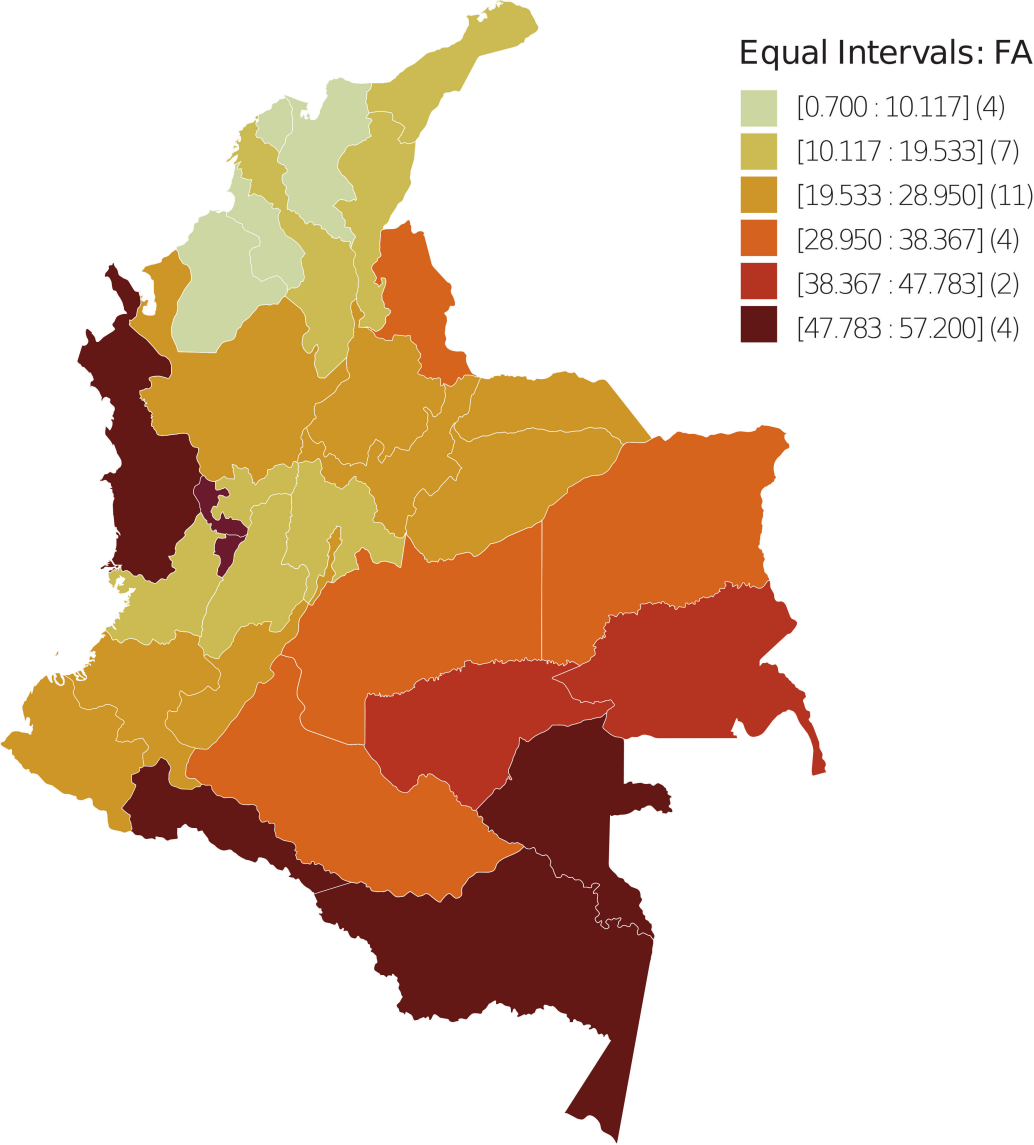
Spatial dispersion of the FA index in Colombia - 2018. The spatial units were divided into six uniform intervals in terms of FA values. Between parentheses is the number of spatial units with FA value in the corresponding interval.

**Figure 2:**
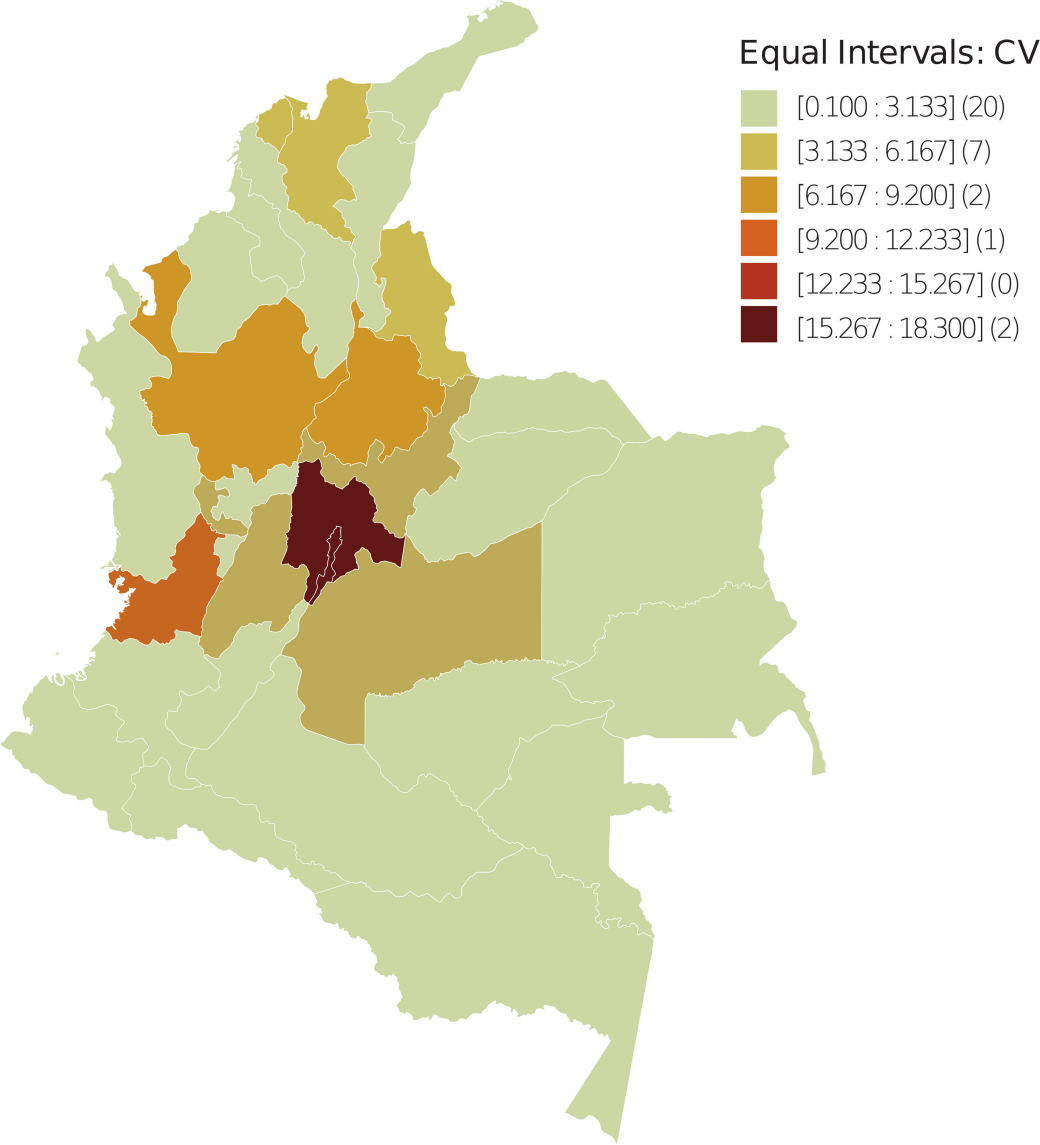
Spatial dispersion of the CV index in Colombia – 2018. The spatial units were divided into six uniform intervals in terms of CV values. Between parentheses is the number of spatial units with CV value in the corresponding interval.

In the departmental distribution of the FA and moving vehicle count, the spatial units were divided into six uniform intervals based on FA and CV values. The number of spatial units with FA and CV values in the corresponding interval is shown in parentheses (**Figures 1**, **2**); data were taken from (57), implementing GeoDA (55).

The spatial distribution observed for the FA variable reveals that from the periphery to the geometrically central areas that coincide with the commercial and productive center of the country, the values range from high to low, which is contrary to the BCM. Meanwhile, the distribution for CV ranges from low to high, which is consistent with the BCM.

**Figure 3:**
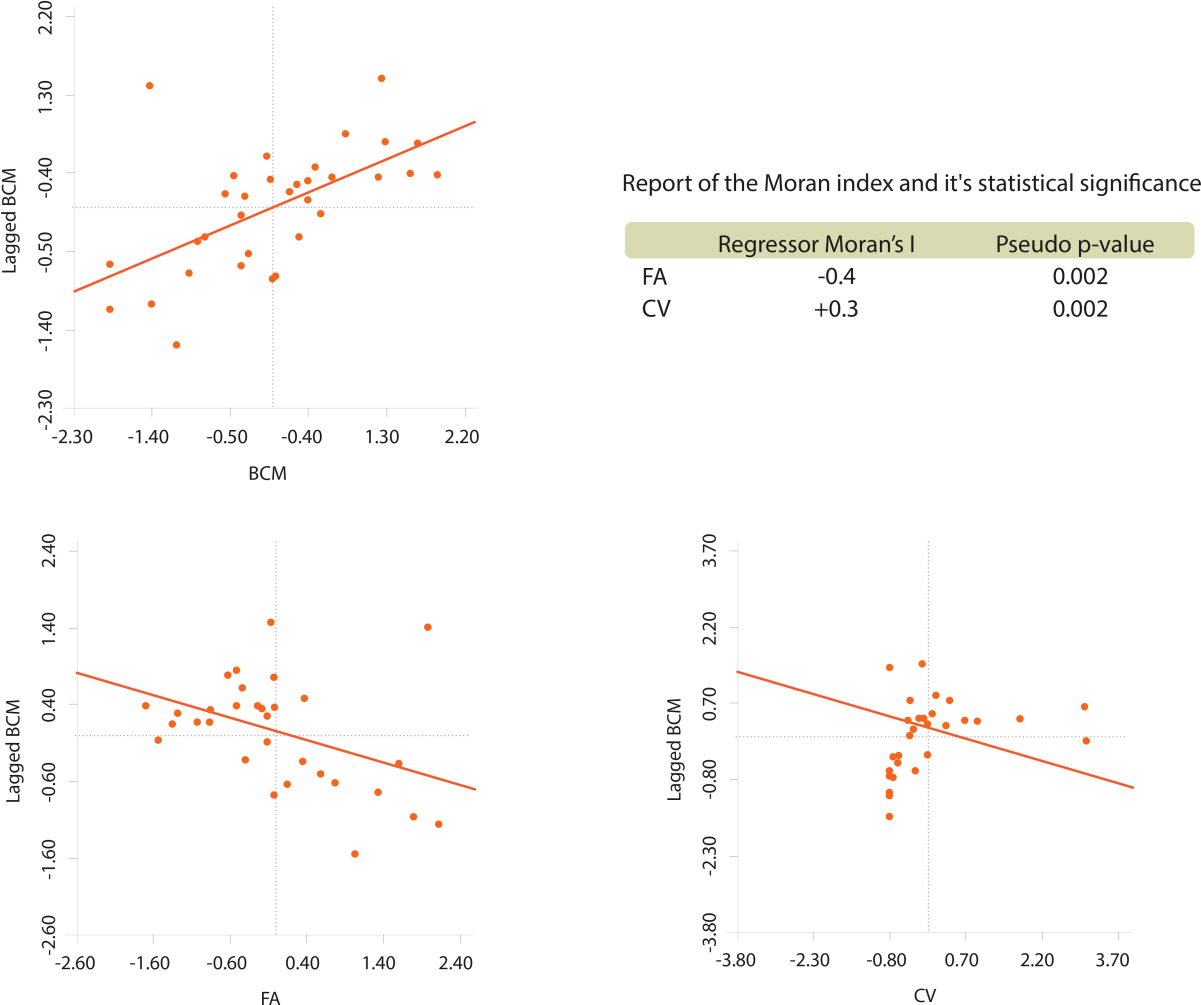
Univariate spatial analysis of BCM and bivariate of W_BCM against each regressor.

To perform a bivariate spatial analysis for BCM spatially lagged for each regressor, the null hypothesis Ho is considered in all correlations: “The distribution of the BCM variable, spatially lagged regarding the regressor, is random”. The decision rule using the Moran index is to reject Ho, if the pseudo p-value is less than 5%. The results of said exploration are presented in **Figure 3**, using a Queen-type neighbourhood matrix. In all the bivariate correlations, there is enough statistical evidence to affirm that there is an aggregation pattern in the BCM distribution spatially lagged regarding each regressor.

Based on these results, we proposed the following behaviour hypothesis between the variables: “BCM varies in space inversely regarding FA and directly in space regarding CV”. The behaviour hypothesis between the variables was contrasted using the classic linear model and two geospatial linear models. It is considered that represents BCM.

The explored models and findings are presented in **Table 3**. The best goodness of fit R^2^ was found using the spatially lagged error term specification. Interestingly, very low multicollinearity (≪30) was observed in the OLS model, which is reasonable given that differential impacts with dummy variables are not being explored. Regarding White’s heteroscedasticity test, the report provides a flattering result in the sense that there is not enough statistical evidence to reject the null hypothesis of homoscedasticity (the p-value is not less than 5%).

**Table 3:** Estimation of OLS-SLM-SEM models.

Models Parameters & statistics	OLS Model y_1_=*a* + *β*_1_ *FA*_1_ + *β*_2_ *CV*_1_ + *μ*_1_	SLM or LAG Models y_1=_ *a* + *β*_1_ *FA*_1_ + *β*_2_ *CV*_1_ + *ρω*_*y*_1_ + *μ*_1 1_	SEM or ERROR Models y_=_ *a* + *β*_1_ *FA*_1_ + *β*_2_ *CV*_1_ + (*1*-*λω*)^-1^ *μ*_1_
*a*	14.3027	9.53939	15.4147
*β*_1_	-0.196189	-0.160553	-0.222462
*β*_2_	0,420776	0.347135	0.333432 **Ж**
Lag coef. *ρ*	N/A	0.374763	N/A
*λ*	N/A	N/A	0.47828
R^2^ adjusted	0.428459	0.556786	0.574534
Prob(F-statistic)	0.00011408	N/A	N/A
Shwarz	192.112	190.85	186.971
Jarque-Bera (Normality test)	Value: 0.6564 Prob.: 0.72023	N/A	N/A
White (Heteroskedasticity test)	Value: 5.8916 Prob.: 0.31691	N/A	N/A
Breusch-Pagan test (Heteroskedasticity)	N/A	Value: 3.1990 Prob.: 0.20200	Value: 3.2511 Prob.: 0.19680
Multicollinearity condition number	4.843880	N/A	N/A
Residuals: Moran’s I	0.291	0.067	0.024
Residuals: Pseudo p-value 999 permutations	0.0050	0.2020	0.3050

OLS, ordinary least squares; SLM, spatial lag model; SEM, spatial autoregressive error model; Ж, Low significance, p=6%.

The OLS model generated good figures, however the LaGrange Likelihood Ratio Test and the Schwartz test generated enough statistical evidence to affirm that a spatial model is better than a classical one. A univariate LISA test was applied to the residuals in each model type with the null hypothesis to be tested, Ho: “The spatial distribution of the residuals is random”. The null hypothesis was rejected whenever the Pseudo p-value was less than 5%. The results are listed in **Table 3**.

When confirming spatial relationships and searching for the best specification for said relationships, we used Vivas-Pacheco (65) as a reference. In the case of the residuals of the OLS model, the hypothesis of randomness was rejected. Since there was a correlation pattern between the residuals the model was discarded as a link between the study variables. Similarly, it was determined that there is not enough statistical evidence to reject the null hypothesis for the spatial lag model (SLM) and the autoregressive spatial error model (SEM). Therefore, both residuals are spatially distributed randomly, making them feasible as a functional link between the variables.

The residuals of the SEM model show a greater tendency towards random spatial distribution since they show closer proximity to zero with the Moran index and less statistical evidence to reject the null hypothesis. However, in this model, there was a low significance for the marginal variation of BCM regarding CV. Therefore, the SEM model is discarded as the best model to relate the variables under study. Consequently, equation 5.


(5) $$\hat{y_{i}}={\left(I_{n}-0.374763\omega \right)}^{-1}\left(9.53939-0.160553FA_{i}+0.347135CV_{i}+\mu_{i}\right)$$

Corresponding to the SLM model, it is the best functional link in the semiotics of the relationships between the variables under study.

## 4 Discussion

This study confirms the conclusions of Díaz-Casas and García-Angulo (35), indicating that the dimensions so far considered as determinants of female BC and classically diffused as ‘‘risk factors’’ of the pathology do not cover but a minority of the incidence.

It’s clear that health entities not only in developing countries but worldwide should not ignore the recommendations of the 2020 report of the American Cancer Society discussed in (36), which mentions the need for further research related to the effect of air quality on morbidity and mortality from cancers other than lung cancer. For example, BC, which is one of the leading causes of female death worldwide. In this regard, the results of this study highlight how the methodological contributions related to the management of spatial data and the inclusion of the environmental dimension as a determinant of BCM, is novel and relevant at the local level and encourages the scientific community to develop this type of research in their countries, having results according to their realities.

More than the realization of a purely spatial mathematical modeling exercise, the results of this research open the discussion that there are more risk factors not considered for BC than those so far disseminated and that one of them is transversal to many other pathologies and is the environmental dimension.

In order to address the problem of this research from a multivariate and ecological perspective; Initially, in the exploratory stage of explanatory variables with spatial lags in relation to BCM, four variables FA, CV, hazardous solid waste index (HSW) (66) and manufacturing industry index (MI) (67) were considered. For statistical significance effects, the variables used were FA and CV.

The present study contributes to sensitizing governmental bodies insofar as the environmental dimension must be considered fundamental in the management models of all economic sectors, including the health sector. Considering environmental dimensions will make it possible to achieve significant differential impacts toward better living conditions in all regions of the national territory.

The cost-benefit balance between allocating resources and efforts to care for the environment versus allocating large amounts of resources to the health sector for costly diagnostic procedures and treatments, could encourage the generation of public policies that strengthen the first factor mentioned without neglecting the second. This strategy represents a conjunctural life saver to which government entities are committed in the face of an avalanche of morbidity and mortality that was previously unexplained until now.

Physical geography can be a limiting factor in an epidemiological dynamic, as well as in an economic one. However, the findings suggest that the figures regarding BCM are relatively low in regions of difficult access, far from large urban centres, with little industrialization and abundant forest. The combination of economic and environmental variables from the perspective of physical geography generates key elements for reflection on an institutional capital that tends to comprehensively improve living conditions in all regions of the national territory.

The phenomenon of the formation of spatial unit clusters that share high BCM and others that share low BCM suggests that economic policies should be generated to not only reduce inequity but also give the environmental dimension of management models the relevance it deserves. The aim would then be to enter a truly sustainable path towards economic development in conditions of equity, fully involving the three dimensions of sustainability throughout the country.

In Colombia there are urban centers that are very difficult to access due to poor or non-existent roads. Due to the above, the possibility of carrying out the study at the level of municipalities or small urban centers was ruled out. If the study could have been carried out at the level of small municipalities, findings with better significance levels could have been found and possibly significance could have been found in other variables, achieving a more realistic model.

## 5 Conclusions

This study evidence how in Colombia the rate BCM is spatially distributed in relation to FA and CV. Based on multivariate methods and the GIS technology software (geospatial data science) with cross-sectional information from the 2018 census, it’s found that the BCM in Colombia is not spatially randomly distributed, but follows cluster aggregation patterns. The results of the present investigation are useful for early warning plans at the epidemiological level to consider factors related to the geographical location of the populations and related environmental elements.

In Colombia, the temporal evolution of the mortality rate for BC in women during the period 2010-2020 shows a growing trend, while in the United States, for example, this trend is decreasing. Factors such as the agility in the provision of services and the level of resources allocated to the health sector could be favorably impacting the rate BCM in developed countries.

The geospatial modeling developed in this study reveals a truly alarming reality: the BCM in Colombia is not distributed randomly but follows patterns of aggregation. This occurs in such a way that departments in geometric, commercial and productive centers form clusters where the rate is higher. On the contrary, in remote or peripheral departments, the index is low. More specifically, the fundamental finding of this research is that the BCM in Colombia is negatively and significantly impacted by the FA, while the impact is positive and significant with respect to vehicular circulation. However, it is important to highlight how these relationships are reinforced when the observations move from spatial units in the peripheries of the country to the central ones.

In Colombia, the environmental dimension mediated by geographic location has been found to be a determinant of the evolution of BCM.

In this study, it’s determined that environmental quality, represented by the FA and CV, are significantly related with BCM.

The scientific evidence based on geospatial data science is conclusive in the sense that the displacement of a woman to a place with better environmental conditions generates a better life expectancy for BC.

In Colombia there is a phenomenon of formation of clusters of spatial units that share high BCM and others that share low BCM. This suggests that the generation of institutional capital to reduce inequity and to give environmental factors in management models the relevance they deserve has been neglected.

## Data availability statement

Publicly available datasets were analyzed in this study. This data can be found here: https://www.dane.gov.co/.

## Author contributions

All authors listed have made a substantial, direct and intellectual contribution to the work, and approved it for publication.

## Supplementary material

Supplementary Figure 1 Evolution BCM in Colombia, 2010-2020

Supplementary Figure 2 Spatial dispersion BCM in Colombia – 2018. The spatial units were divided into six uniform intervals in terms of BCMR values. Between parentheses is the number of spatial units with BCM value in the corresponding interval

Supplementary Table 1 Common air pollutants that affect health, grouped by origin.

Supplementary Table 2 Mortality of the main types of cancer worldwide in 2018.

Supplementary Table 3 Mortality rate for breast cancer in Colombia, 2010-2020.

Supplementary Table 4 Cross-section data year 2018, explored.
