# Supplementary figures and images for: Multivariate analysis in data science for the geospatial distribution of the breast cancer mortality rate in Colombia
Source: Front Oncol. 2023 Jan 6;12:1055655. doi: 10.3389/fonc.2022.1055655 (PMC9853892; doi:10.3389/fonc.2022.1055655)

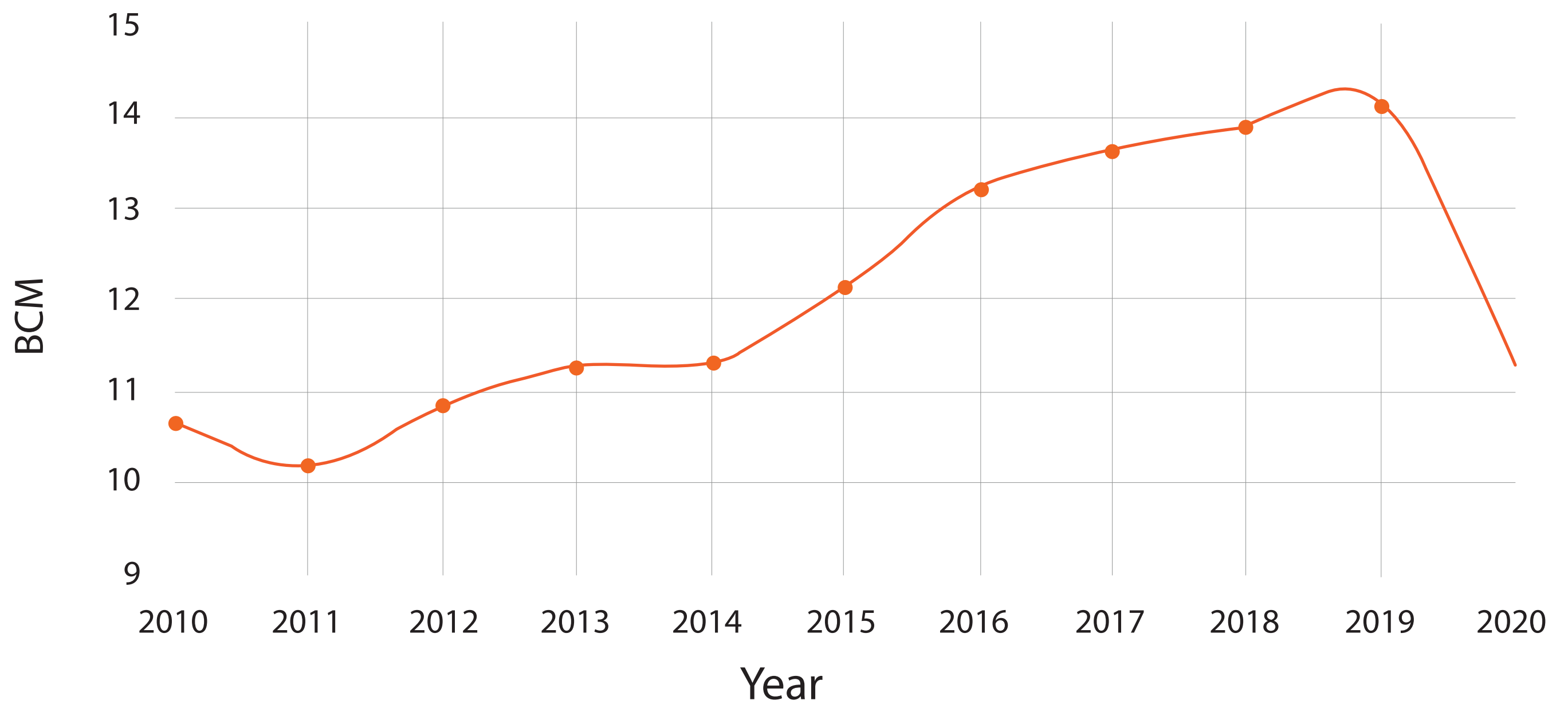

Supplement: Supplementary Figure 1 — Evolution BCM in Colombia, 2010-2020 [file Image_1.pdf]

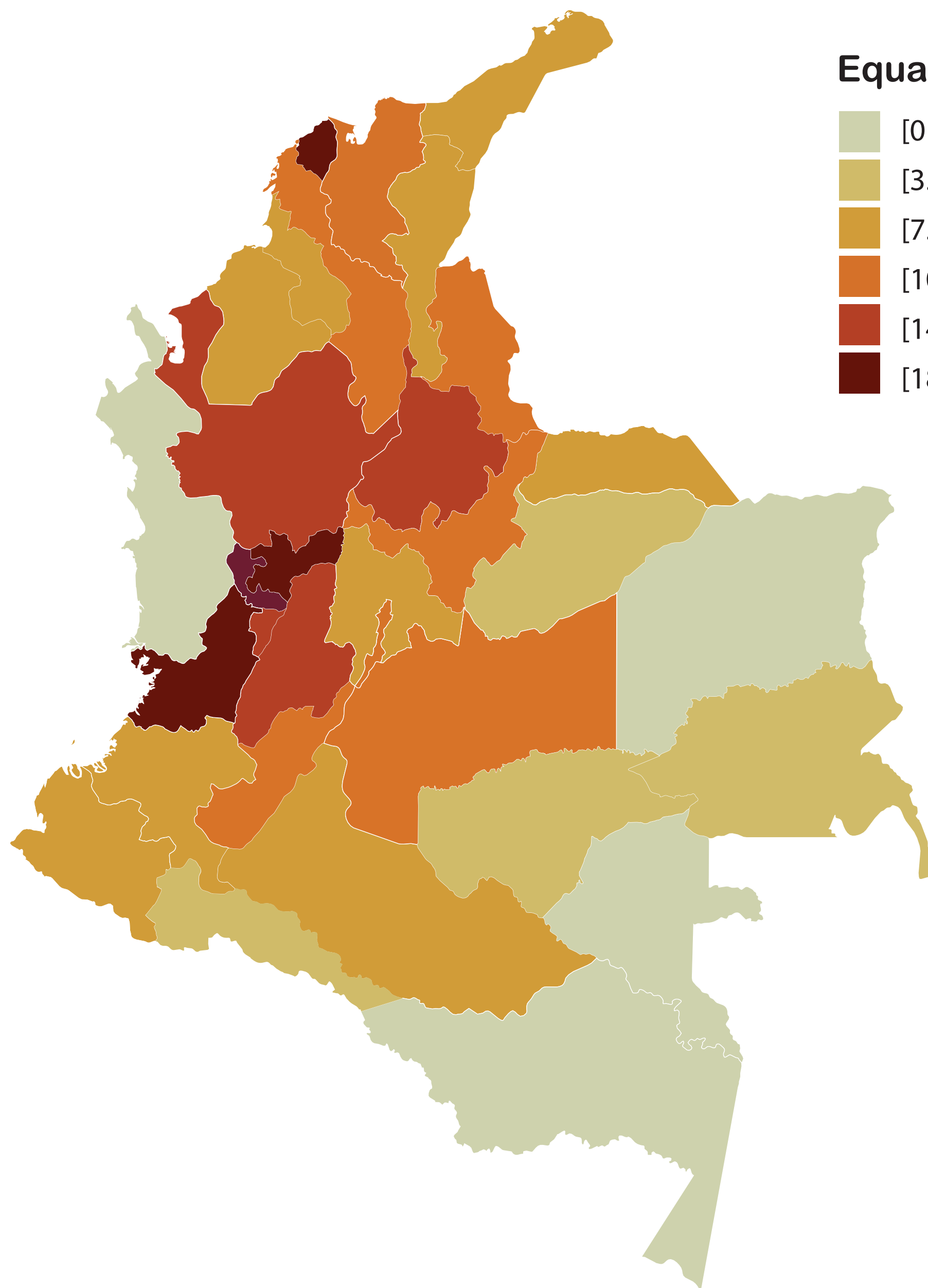

## Equal Intervals: BCM

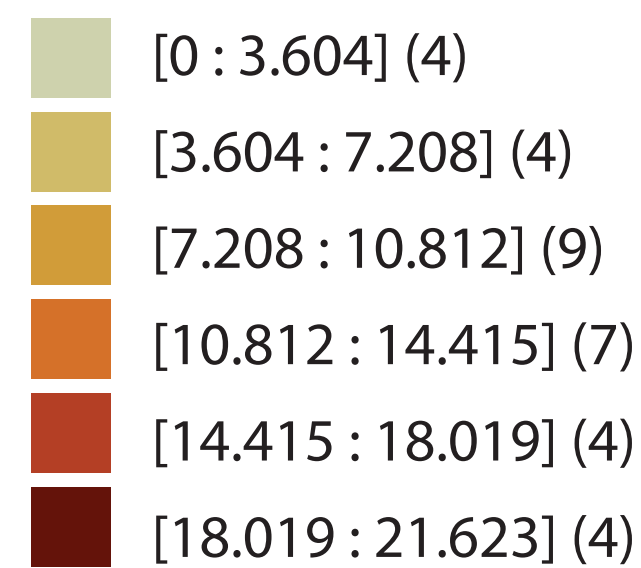

Supplement: Supplementary Figure 2 — Spatial dispersion BCM in Colombia – 2018. The spatial units were divided into six uniform intervals in terms of BCMR values. Between parentheses is the number of spatial units with BCM value in the corresponding interval [file Image_2.pdf]
