# Supplementary material for: Multivariate analysis in data science for the geospatial distribution of the breast cancer mortality rate in Colombia
Source: Front Oncol. 2023 Jan 6;12:1055655. doi: 10.3389/fonc.2022.1055655 (PMC9853892; doi:10.3389/fonc.2022.1055655)
Supplement: Supplementary Table 1 — Common air pollutants that affect health, grouped by origin. [file Table_1.docx]

Supplementary Table 1. Common air pollutants that affect health, grouped by origin.

| **Predominantly outdoor air pollutants** | |
| --- | --- |
| Ozone (O_3_ ) | Generated through photochemical reactions in the atmosphere from nitrogen oxides (NOx) and volatile organic compounds (VOCs), as well as natural processes (eg, stratosphere). |
| Arsenic (As), Chromium (Cr) | Fine particulate matter from coal combustion (particulate matter (PM) 2.5). |
| Nickel (Ni), Vanadium (V) | Combustion of residual fine PM oil (PM 2.5). |
| **Predominantly Indoor Air Pollutants** | |
| Radon | Building materials (concrete, stone), groundwater. |
| Asbestos, minerals, synthetic fibers | Fireproof, acoustic, thermal or electrical insulation. |
| Biological contaminant | Infections, dust mites, animal dander, allergens. |
| **Indoor and outdoor air pollutants, suspended PM** | |
| Good PM (PM 2.5) | - Outdoors: combustion of fossil fuels, conversion of gas to particles, burning of biomass. - Indoor: combustion of biomass fuel, smoking. |
| Thick PM (PM 2,5-10,0) | - Outdoors: dust storms, windblown soil, pollen. - Interior: mold spores, resuspended dust. |
| Nitrogen Dioxide (NO_2_) | - Exterior: combustion of fossil fuels (diesel vehicle emissions). - Interior: smoking tobacco, gas stoves. |
| Volatile Organic Compounds (VOCs) | - Exterior: petrochemical solvents, evaporated fuels, biogenics. - Interior: fuel and paint fumes, combustion, adhesives, cosmetics, solvents, particle board (formaldehyde), insulation, furniture, tobacco smoke. |
| Carbon Monoxide (CO) | - Outdoors: fossil fuel combustion, biomass burning, forest fires. - Interior: tobacco smoke, unvented gas heaters. |
| Lead (Pb) | - Outdoors: industrial emissions, leaded fuel combustion, lead processing. - Interior: Lead paint wear. |
| Mercury (Hg) | - Outside: coal combustion, mineral refining. - Interior: Fungicides in paints, broken thermometers, ritual use. |
| Pesticides | - Exterior: agricultural. - Interior: home applications of herbicides, insecticides, fungicides, etc. |
| Ammonia | - Outside: cattle yards. - Interior: metabolic activity, cleaning products. |
| Hazardous Air Pollutants (HAPs) (eg, benzene, 1,3-butadiene, formaldehyde, acids) | - Exterior: incomplete combustion, chemical processing. - Interior: use of solvents. |
