# Supplementary material for: Multivariate analysis in data science for the geospatial distribution of the breast cancer mortality rate in Colombia
Source: Front Oncol. 2023 Jan 6;12:1055655. doi: 10.3389/fonc.2022.1055655 (PMC9853892; doi:10.3389/fonc.2022.1055655)
Supplement: Supplementary Table 2 — Mortality of the main types of cancer worldwide in 2018. [file Table_2.docx]

Supplementary Table 2. Mortality of the main types of cancer worldwide in 2018.

| Type of cancer | # deaths | % deaths |
| --- | --- | --- |
| Lung | 1,761,007 | 18.4 |
| Stomach | 782,685 | 8.2 |
| Liver | 781,631 | 8.1 |
| Breast | 626,679 | 6.6 |
| Colon | 551,269 | 5.8 |
| Esophagus | 508,585 | 5.3 |
| Pancreas | 432,242 | 4.5 |
| Prostate | 358,989 | 3.8 |
| Cervix | 311,365 | 3.3 |
| Rectum | 310,394 | 3.2 |
| 26 other types of cancer | 6,424,846 | 67.2 |
