# Supplementary material for: Multivariate analysis in data science for the geospatial distribution of the breast cancer mortality rate in Colombia
Source: Front Oncol. 2023 Jan 6;12:1055655. doi: 10.3389/fonc.2022.1055655 (PMC9853892; doi:10.3389/fonc.2022.1055655)
Supplement: Supplementary Table 3 — Mortality rate for breast cancer in Colombia, 2010-2020. [file Table_3.docx]

Supplementary Table 3. Mortality rate for breast cancer in Colombia, 2010-2020.

| **Year** | **Total mortality** | **Female mortality** | **Female mortality from breast cancer** | **Population of Colombia** | **Female population** | **BCM** |
| --- | --- | --- | --- | --- | --- | --- |
| 2010 | 200,524 | 86.250 | 2,394 | 44,349,775 | 22,486,954 | 10.6 |
| 2011 | 195,823 | 84.590 | 2,319 | 44,796,093 | 22,726,456 | 10.2 |
| 2012 | 199,756 | 86.654 | 2,489 | 45,217,714 | 22,955,760 | 10.8 |
| 2013 | 203,071 | 88.355 | 2,613 | 45,622,930 | 23,178,743 | 11.3 |
| 2014 | 201,812 | 90.168 | 2,647 | 46,021.270 | 23,401,956 | 11.3 |
| 2015 | 219,472 | 98.037 | 2,865 | 46.431,100 | 23,636,050 | 12.1 |
| 2016 | 223,078 | 99.242 | 3,157 | 46,900,058 | 23,911,986 | 13.2 |
| 2017 | 227,624 | 102.119 | 3,300 | 47,407,570 | 24,222,239 | 13.6 |
| 2018 | 236,932 | 106.075 | 3,428 | 48,258,494 | 24,685,207 | 13.9 |
| 2019 | 243,396 | 109.230 | 3,563 | 49,395,678 | 25,271,995 | 14.1 |
| 2020 | 238,209 | 102.767 | 2,909 | 50,372,424 | 25,777,542 | 11.3 |
