# Supplementary material for: Multivariate analysis in data science for the geospatial distribution of the breast cancer mortality rate in Colombia
Source: Front Oncol. 2023 Jan 6;12:1055655. doi: 10.3389/fonc.2022.1055655 (PMC9853892; doi:10.3389/fonc.2022.1055655)
Supplement: Supplementary Table 4 — Cross-section data year 2018, explored. [file Table_4.docx]

Supplementary Table 4. Cross-section data year 2018, explored.

| ID | Departments | Population | Vehicle  registration | BCM | FA | CV | MI | HSW |
| --- | --- | --- | --- | --- | --- | --- | --- | --- |
| 05 | Antioquia | 6,691,030 | 298,930 | 14.5 | 25.7 | 8.2 | 19.1 | 12.2 |
| 08 | Atlántico | 2,545,924 | 118,339 | 19.8 | 0.7 | 3.3 | 5.8 | 6.4 |
| 11 | Bogotá, D.C | 8,181,047 | 496,385 | 13.9 | 24.2 | 13.6 | 18.2 | 6.4 |
| 13 | Bolívar | 2,171,280 | 89,241 | 11.8 | 13.2 | 2.5 | 5.0 | 7.0 |
| 15 | Boyacá | 1,282,063 | 193,860 | 12.3 | 24.2 | 5.3 | 2.7 | 2.7 |
| 17 | Caldas | 993,866 | 72,460 | 18.1 | 16.7 | 2.0 | 1.7 | 0.9 |
| 18 | Caquetá | 496,241 | 28,986 | 9.1 | 34.6 | 0.8 | 0.1 | 0 |
| 19 | Cauca | 1,415,933 | 97,405 | 8.1 | 22.6 | 2.6 | 2.6 | 0.7 |
| 20 | Cesar | 1,065,673 | 66,671 | 10.6 | 13.2 | 1.8 | 0.5 | 2.3 |
| 23 | Córdoba | 1,788,507 | 83,362 | 8.9 | 6.1 | 2.3 | 1.5 | 0.4 |
| 25 | Cundinamarca | 2,804,238 | 170,147 | 10.3 | 19.2 | 4.7 | 10.9 | 4.5 |
| 27 | Chocó | 515,145 | 3,895 | 2.6 | 55.3 | 0.1 | 0 | 0.0 |
| 41 | Huila | 1,197,081 | 71,369 | 13.1 | 23.2 | 2.0 | 0.5 | 3.3 |
| 44 | La Guajira | 1,040,157 | 84,709 | 7.6 | 10.6 | 2.3 | 0.1 | 0.9 |
| 47 | Magdalena | 1,298,691 | 126,869 | 13.0 | 7.0 | 3.5 | 0.5 | 0.7 |
| 50 | Meta | 1,016,701 | 125,393 | 12.5 | 31.0 | 3.5 | 0.7 | 10.4 |
| 52 | Nariño | 1,809,116 | 88,503 | 8.6 | 28.1 | 2.4 | 0.3 | 0.3 |
| 54 | Norte de Santander | 1,391,239 | 149,347 | 13.5 | 31.4 | 4.1 | 0.8 | 1.7 |
| 63 | Quindío | 575,010 | 108,600 | 17.9 | 24.8 | 3.0 | 0.3 | 0.2 |
| 66 | Risaralda | 967,767 | 201,775 | 20.3 | 25.6 | 5.5 | 1.8 | 0.4 |
| 68 | Santander | 2,090,839 | 252,992 | 17.7 | 23.1 | 7.0 | 10.7 | 16.1 |
| 70 | Sucre | 877,057 | 34,976 | 8.7 | 3.1 | 1.0 | 0.5 | 0.5 |
| 73 | Tolima | 1,419,947 | 158,804 | 15.5 | 18.2 | 4.4 | 1.8 | 1.3 |
| 76 | Valle del Cauca | 4,756,113 | 443,937 | 21.6 | 18.4 | 12.2 | 13.5 | 7.9 |
| 81 | Arauca | 270,708 | 11,425 | 10.8 | 25.7 | 0.3 | 0.1 | 1.6 |
| 85 | Casanare | 375,249 | 27,649 | 6.3 | 20.2 | 0.7 | 0.3 | 7.2 |
| 86 | Putumayo | 358,896 | 13,136 | 5.8 | 49.5 | 0.4 | 0 | 4.0 |
| 91 | Amazonas | 78,830 | 3,327 | 2.7 | 52.4 | 0.1 | 0 | 0 |
| 94 | Guainía | 43,446 | 1,834 | 4.4 | 41.2 | 0.1 | 0 | 0 |
| 95 | Guaviare | 115,829 | 4,888 | 5.2 | 45.4 | 0.1 | 0 | 0 |
| 97 | Vaupés | 44,928 | 1,896 | 0 | 57.2 | 0.1 | 0 | 0 |
| 99 | Vichada | 77,276 | 3,261 | 0 | 37.4 | 0.1 | 0 | 0 |

Population (15), vehicle registration (58), BCM (63), MI (67), HSW (66).

Second part: origin of statistical information and sources.

| **Source** | **Statistical Information** |
| --- | --- |
| **DANE** | Population by departments - census statistics 2018. Female deaths from breast cancer by department - vital statistics. |
| **(INVIAS, acronym in Spanish)** | CV– circulating vehicle index and vehicle registration |
| **(SIGOT-IGAC, acronym in Spanish)** | FA – forest area index |
| **(IDEAM, acronym in Spanish)** | HSW – hazardous solid waste index |

DANE (15), INVIAS (59), SIGOT-IGAC (57), IDEAM (37) – Republica de Colombia.
